# Computational prediction of structural, electronic, and optical properties and phase stability of double perovskites K_2_SnX_6_ (X = I, Br, Cl)†

**DOI:** 10.1039/c9ra09232c

**Published:** 2019-12-24

**Authors:** Un-Gi Jong, Chol-Jun Yu, Yun-Hyok Kye

**Affiliations:** Chair of Computational Materials Design, Faculty of Materials Science, Kim Il Sung University Ryongnam-Dong, Taesong District Pyongyang Democratic People’s Republic of Korea cj.yu@ryongnamsan.edu.kp; Natural Science Centre, Kim Il Sung University Ryongnam-Dong, Taesong District Pyongyang Democratic People’s Republic of Korea ug.jong@ryongnamsan.edu.kp

## Abstract

The vacancy-ordered double perovskites K_2_SnX_6_ (X = I, Br, Cl) attract significant research interest due to their potential applications as light absorbing materials in perovskite solar cells. However, deeper insight into their material properties at the atomic scale is currently lacking. Here we present a systematic investigation of the structural, electronic, and optical properties and phase stabilities of the cubic, tetragonal, and monoclinic phases based on density functional theory calculations. Quantitatively reliable predictions of lattice constants, band gaps, effective masses of charge carriers, and exciton binding energies are provided and compared with the available experimental data, revealing the tendency of the band gap and exciton binding energy to increase on lowering the crystallographic symmetry from cubic to monoclinic and on moving from I to Cl. We highlight that cubic K_2_SnBr_6_ and monoclinic K_2_SnI_6_ are suitable for applications as light absorbers for solar cell devices due to their appropriate band gaps of 1.65 and 1.16 eV and low exciton binding energies of 59.4 and 15.3 meV, respectively. The constant-volume Helmholtz free energies are determined through phonon calculations, which predict phase transition temperatures of 449, 433 and 281 K for cubic–tetragonal and 345, 301 and 210 K for tetragonal–monoclinic transitions for X = I, Br and Cl, respectively. Our calculations provide an understanding of the material properties of the vacancy-ordered double perovskites K_2_SnX_6_, which could help in devising a low-cost and high performance perovskite solar cell.

## Abstract 2

HSE + SOC were used to calculate the band structures of the cubic, tetragonal, and monoclinic phases of the double perovskites K_2_SnX_6_ (X = I, Br, Cl).

## Graphical abstract

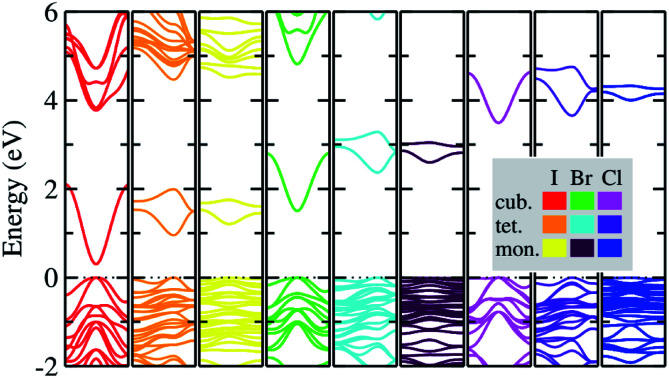

## 1 Introduction

During the past decade, organic–inorganic hybrid halide perovskites have revolutionized the field of photovoltaics due to rapid improvements in power conversion efficiency (PCE) and their significantly lower fabrication cost compared with traditional silicon solar cells.^1,2^ Prototype perovskite solar cells (PSCs) usually adopt methylammonium lead iodide (CH_3_NH_3_PbI_3_ or MAPI_3_) as a light absorbing material, since it is composed of Earth abundant elements and exhibits unique properties that are beneficial to solar cell applications.^3–9^ Using this type of hybrid halide perovskite, the PCE reached a certified record of 25.2%,^10^ which is high enough for solar cell applications, but there are also fatal problems, namely their instability to humidity, temperature and light and moreover the toxicity of lead, which still hinder the commercialization of PSCs.^6,11–15^

These challenges could be partially addressed by utilizing solid solutions through mixing Br or Cl with I anions^11,16–18^ and mixing another organic moiety such as formamidinium (FA) or even inorganic Cs and Rb with MA cations. Noh *et al.*^11^ demonstrated that the stability of the mixed-halide perovskite MAPb(I_1−*x*_Br_*x*_)_3_ was significantly improved with relatively high PCEs for mixing ratio *x* < 0.2, and a similar effect was found on partially replacing I with Cl in MAPb(I_1−*x*_Cl_*x*_)_3_.^19–21^ Alternatively, double or triple mixed-cation perovskites have been found to have substantially improved efficiencies and phase stabilities.^22–27^ For instance, Niu *et al.*^22^ fabricated PSCs with a composition of Cs_*x*_MA_1−*x*_PbI_3_, reporting that introducing a small amount of Cs (*x* ∼ 0.09) resulted in not only better stability but also higher efficiency of solar cells. By using a triple mixture of Cs/MA/FA cations, Saliba *et al.*^25^ further achieved higher peak efficiencies of 21.1% and 18% after 250 hours under operational conditions. On the other hand, mixed-cation and mixed-halide perovskites like Cs_*y*_FA_1−*y*_Pb(Br_*x*_I_1−*x*_)_3_^24^ were also examined in order to tune the phase stability, photostability and optoelectronic properties by carefully changing the chemical compositions of cations (*y*) and halide anions (*x*).

In parallel with these investigations on hybrid organic–inorganic halide perovskites, fully replacing the organic cations with the inorganic Cs or Rb cations, resulting in all-inorganic perovskites, has been regarded as a promising way to improve the stability because of the lower sensitivity of inorganic cations to moisture.^28–34^ In fact, many papers reported that PSCs containing the inorganic cesium lead iodide perovskite (CsPbI_3_) exhibited a high PCE comparable to those of hybrid PSCs and significantly enhanced device stability.^28,30,33^ However, it is challenging to form the photoactive black phase of CsPbI_3_ with a cubic lattice (α-CsPbI_3_) at room temperature,^30,35^ and moreover it still contains toxic lead. Accordingly, CsSnI_3_ has been suggested as a non-hygroscopic and non-toxic halide perovskite. Several groups have synthesized the photoactive cubic CsSnI_3_, which is stable at room temperature, but CsSnI_3_-based PSCs have shown too low PCEs of up to 2%.^36–38^ Theoretical and experimental investigations suggested that the poor efficiency is due to the ready oxidation of tin cations from the Sn^2+^ state to the Sn^4+^ state, resulting in a deterioration of the optoelectronic properties of CsSnI_3_.^39,40^

The structural diversity of perovskite materials can open an outlet for avoiding this oxidation. For example, the vacancy-ordered double perovskite Cs_2_SnI_6_ is obtained by removing every other Sn cation from the fully occupied conventional perovskite CsSnI_3_. It was reported that Cs_2_SnI_6_ adopts the cubic phase at room temperature with a direct band gap of about 1.3 eV, strong visible light absorption coefficients, long carrier mobilities, and air and moisture stability, all of which are advantageous for solar cell applications.^41–45^ Despite these merits, unfortunately there is an obstacle preventing the wide application of Cs_2_SnI_6_ in large-scale and low-cost PSCs, namely the small amount of cesium in the Earth’s crust. In fact, cesium occupies only 0.00019% of the Earth’s crust by weight, and is considered to be the 50th most common element in the periodic table. This motivated researchers to use potassium instead, which is in the same group as cesium and is the 7th most common element, occupying 2.6% of the Earth’s crust by weight, which is 10 000 times larger than the amount of cesium. Therefore, replacing cesium with potassium satisfies the criteria for realizing stable, environmentally friendly, large-scale and low-cost all-inorganic PSCs through the utilization of Earth abundant elements. Back in the late 1970s, only a few investigations focused on the structural properties of K_2_SnBr_6_ and K_2_SnCl_6_,^46,47^ and thus, comprehensive research on the potassium tin halide vacancy-ordered double perovskites K_2_SnX_6_ (X = I, Br, Cl) is indispensable for their photovoltaic applications.

In the present work, we perform density functional theory (DFT) calculations to explore the structural, electronic, and optical properties and phase stability of the vacancy-ordered double perovskites K_2_SnX_6_ (X = I, Br, Cl), aiming to investigate the possibility of their solar cell applications. Keeping in mind that vacancy-ordered double perovskites generally undergo a series of phase transitions from the monoclinic to tetragonal, and then to the cubic phase upon increasing the temperature, we begin by optimizing the crystal structures of K_2_SnX_6_ in the cubic (space group *Fm*3̄*m*), tetragonal (*P*4/*mnc*) and monoclinic (*P*2_1_/*n*) phases. Using these optimized structural parameters, we calculate the electronic and optical properties including electronic energy bands with density of states (DOS), effective masses of charge carriers, dielectric constants, exciton binding energies and light absorption coefficients, providing a systematic comparison of these properties on changing the halogen atom and crystalline symmetry. Finally, by using the density functional perturbation theory (DFPT) method, we determine the phonon dispersion curves with phonon DOS, and estimate the phase transition temperatures for the cubic to tetragonal and tetragonal to monoclinic phase transitions based on the obtained constant-volume Helmholtz free energies.

## 2 Computational methods

All calculations were performed using the norm-conserving pseudopotential (NCPP) plane wave method as implemented in the ABINIT package.^48^ We generated the Troullier–Martins type soft NCPPs using the FHI98PP code^49^ to describe the interactions between the ions and valence electrons, using the valence electronic configurations: Cs – 5s^2^5p^6^6s^1^, Cl – 3s^2^3p^5^, Br – 4s^2^4p^5^, I – 5s^2^5p^5^, and Sn – 5s^2^5p^2^. We employed the Perdew–Burke–Ernzerhof functional (PBE)^50^ within the generalized gradient approximation (GGA) for the exchange–correlation interactions between the valence electrons. The cutoff energy for plane wave basis sets and the Monkhorst–Pack *k*-points for electron density were set to be 40 Ha and (6 × 6 × 6) for cubic and (6 × 6 × 4) for tetragonal and monoclinic phases, giving a total energy accuracy of 5 meV per formula unit. The variable-cell structural optimizations were performed until the forces acting on the atoms were less than 10^−5^ Ha Bohr^−1^ with a tight self-consistent convergence threshold of 10^−14^ Ha for the total energy.

In order to obtain a reliable description of the electronic structures, we calculated the electronic band structures using the PBE and the Heyd–Scuseria–Ernzerhof (HSE06) hybrid functionals^51^ with and without the spin–orbit coupling (SOC) effect. We replaced 20% of the PBE exchange functional with the exact Hartree–Fock exchange functional, producing energy band gaps in good agreement with the experimental values for halide perovskites,^52,53^ and we considered the SOC effect only when calculating the electronic structures. The optoelectronic properties including frequency-dependent dielectric functions, light absorption coefficients, effective masses of electrons and holes, and exciton binding energies were estimated using the computational methods detailed in our previous work.^18,21,52^ In particular, the effective masses of electrons and holes were estimated within the parabolic approximation, using the refined energy band structures around the conduction band minimum and valence band maximum which were calculated with finer *k*-points of (10 × 10 × 10) for the cubic phase and (10 × 10 × 8) for the tetragonal and monoclinic phases.

To assess the phase stability of K_2_SnX_6_, we calculated the phonon dispersions and phonon DOS using the DFPT method as implemented in the ABINIT package with a tighter convergence threshold of 10^−18^ for potential residual. When calculating the phonon DOS, we used thermal broadening with a smearing parameter of 0.05 Ha in order to improve the convergence. By post-processing the calculated phonon DOS, we evaluated the constant-volume Helmholtz free energies of the cubic, tetragonal and monoclinic phases of K_2_SnX_6_ on increasing the temperature from 0 to 1000 K with intervals of 10 K. To obtain more reliable phonon DOS and Helmholtz free energies, finer meshes of (80 × 80 × 80) for cubic and (100 × 100 × 80) for tetragonal and monoclinic phases were used. With the calculated Helmholtz free energies, we calculated the temperatures of the phase transitions from one phase to another phase using the free energy differences.

## 3 Results and discussion

### 3.1 Crystal structures

Vacancy-ordered double perovskites are generally regarded to successively adopt cubic, tetragonal, and monoclinic phases upon decreasing temperature. In fact, the cubic, tetragonal, and monoclinic phases of K_2_SnCl_6_ and K_2_SnBr_6_ were experimentally found to stabilize at different temperatures.^46,47^ Thus, as a first step, we performed optimizations of the K_2_SnX_6_ (X = I, Br, Cl) crystal structures in the cubic (*Fm*3̄*m*), tetragonal (*P*4/*mnc*) and monoclinic (*P*2_1_/*n*) phases, as shown in Fig. S1.†Table 1 presents the Goldschmidt’s tolerance factors (*t*_G_), octahedral factors (*t*_o_), radius ratios (*t*_r_), lattice constants (*a*, *b*, *c*) and angles (*β*) calculated using the PBE-GGA functional in comparison with the available experimental data^46,47^ for K_2_SnX_6_ (X = I, Br, Cl).

**Table 1:** Goldschmidt’s tolerance factors (*t*_G_), octahedral factors (*t*_o_), radius ratios (*t*_r_), lattice constants (*a*, *b*, *c*) and angles (*β*) of K_2_SnX_6_ (X = I, Br, Cl) in the cubic, tetragonal, and monoclinic phases calculated using the PBE functional

Material	Phase	*t*_G_	*t*_o_	*t*_r_	Cal.	Exp.^46,47^
K_2_SnI_6_	Cub.	0.85	0.32	0.79	*a* = 11.66 (Å)	—
	Tet.				*a* = 8.25, *b* = 11.76 (Å)	—
	Mono.				*a* = 8.29, *b* = 8.32, *c* = 11.69 (Å)	—
					*β* = 90.25 (deg.)	—
K_2_SnBr_6_	Cub.	0.87	0.36	0.85	*a* = 10.51 (Å)	*a* = 10.48 (Å)
	Tet.				*a* = 7.50, *b* = 10.67 (Å)	—
	Mono.				*a* = 7.45, *b* = 7.47, *c* = 10.68 (Å)	*a* = 7.43, *b* = 7.44, *c* = 10.62 (Å)
					*β* = 90.17 (deg.)	*β* = 90.18 (deg.)
K_2_SnCl_6_	Cub.	0.88	0.39	0.89	*a* = 10.02 (Å)	*a* = 9.99 (Å)
	Tet.				*a* = 7.09, *b* = 10.01 (Å)	*a* = 7.06, *b* = 9.98 (Å)
	Mono.				*a* = 7.07, *b* = 7.05, *c* = 10.03 (Å)	*a* = 7.02, *b* = 7.01, *c* = 9.99 (Å)
					*β* = 90.11 (deg.)	*β* = 90.13 (deg.)

As for the conventional perovskite ABX_3_, we assessed the formability of the perovskite structure in K_2_SnX_6_, simply by using the Goldschmidt tolerance factor, 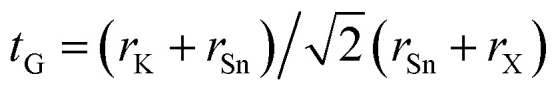, where *r*_K_, *r*_Sn_, and *r*_X_ are the Shannon ionic radii for K^+^, Sn^4+^, and X^−^ ions, respectively. Based on the fact that a tolerance factor within the range of 0.8 < *t*_G_ < 1.0 allows the formation of the perovskite structure, we can expect that all three K_2_SnX_6_ (X = I, Br, Cl) compounds crystallize in the cubic perovskite phase due to their suitable tolerance factors of 0.88, 0.87, and 0.85 (Table 1). On the other hand, Cai *et al.*^54^ used the octahedral factor, *t*_o_ = *r*_B_/*r*_X_, and radius ratio, *t*_r_ = *r*_A_/(*D*_XX_ − *r*_X_), to empirically predict the formation and distortion of the crystalline structure in vacancy-ordered double perovskites A_2_BX_6_, where *D*_XX_ is the nearest neighbor X–X bond length as calculated for the cubic phase. According to their survey of experimentally known A_2_BX_6_ compounds, a smaller *t*_o_ disfavors the formation of BX_6_ octahedra, while a smaller *t*_r_ favors the distortion of octahedra, lowering the symmetry of the crystal structure. When the octahedral factor is within the range 0.29 < *t*_o_ < 0.55 and the radius ratio is within 0.87 < *t*_r_ < 1.00, A_2_BX_6_ stabilizes in the cubic phase at room temperature. As listed in Table 1, the octahedral factor and radius ratio decrease on going from X = Cl to X = I, implying that as the ionic radius of the halogen anion increases, the perovskite structure undergoes octahedral tilting and accordingly its symmetry is lowered from the cubic structure to a lower-symmetry structure at room temperature. From the calculated octahedral factor *t*_o_ = 0.39 and radius ratio *t*_r_ = 0.89 for K_2_SnCl_6_, it can be concluded that it crystallizes in the stable cubic phase at room temperature, as confirmed in previous experiments.^46^ It should be noted that although such considerations of the structural factors of *t*_G_, *t*_o_, and *t*_r_ could provide a qualitative prediction of the formation of the perovskite structure and octahedral distortion, a quantitative description of phase stability should be based on lattice dynamics calculations.

Regarding the lattice constants, the PBE functional slightly overestimated them compared with the experimental data,^46,47^ with relative errors of less than 1% for the cubic, tetragonal, and monoclinic phases of K_2_SnCl_6_ and K_2_SnBr_6_. The calculated lattice angles for the monoclinic phases were in good agreement with the experimental values,^46,47^ with relative errors of less than 0.3% (Table 1). As the ionic radius of the halogen anions increases, the lattice constants of all the phases increase and the lattice angle of the monoclinic phase deviates significantly from 90°, indicating that the octahedral distortions become even more pronounced on going from X = Cl to X = Br to X = I. This can be attributed to the weakening of chemical bonds between the Sn and X atoms, which subsequently increases the bond lengths and distorts the octahedra. These trends in the crystal parameters coincide with the well-known fact that perovskites with smaller octahedral factors tend to form non-cubic structures with more distorted octahedra at room temperature. Although there is a lack of experimental data for all the phases of K_2_SnI_6_, we can expect that our work provides a reliable prediction for those.

### 3.2 Electronic structures

**Fig. 1:**
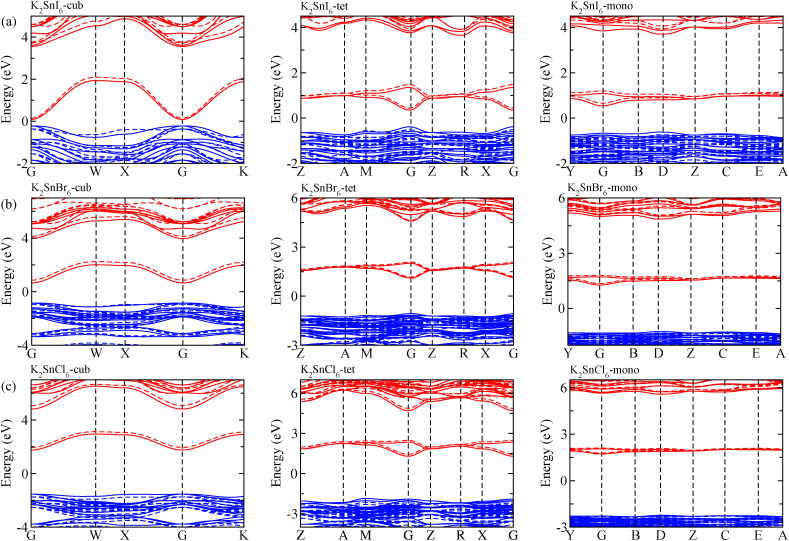
Electronic band structures of (a) K_2_SnI_6_, (b) K_2_SnBr_6_ and (c) K_2_SnCl_6_ in the cubic (left), tetragonal (middle) and monoclinic (right) phases, calculated using the HSE06 hybrid functional with (solid line) and without (dashed line) spin–orbit coupling. Blue and red colors indicate the valence and conduction bands, respectively.

The electronic properties including the energy band structures and DOS can be used to predict the performance of solar cells through estimation of the light absorption capability. Thus, we calculated them, together with the charge densities corresponding to the valence band maximum (VBM) and the conduction band minimum (CBM), using the PBE-GGA and hybrid HSE06 functionals with and without the spin–orbit coupling (SOC) effect for all the phases of K_2_SnX_6_ (X = I, Br, Cl). Fig. 1 shows the energy band structures calculated using the HSE06 hybrid functional with and without the SOC effect for each phase of K_2_SnX_6_ (X = I, Br, Cl). For the cubic and tetragonal phases, all the perovskite compounds were found to have direct band gaps at the gamma point (G) of the Brillouin zone (BZ), and these characteristics of the direct band gap are consistent with previous DFT calculations on other types of vacancy-ordered double perovskites such as Cs_2_SnI_6_ and Rb_2_SnI_6_.^44,54^ On the other hand, in the monoclinic phase all the compounds were predicted to have indirect band gaps between the CBM at the G point and the VBM at the D point of the BZ. Interestingly, the energy band corresponding to the VBM is almost dispersionless around the G point for the cubic and tetragonal phases and the D point for the monoclinic phase, and from the flatness of the valence band, we can expect that the effective masses of the holes are much larger than those of the electrons.

In Table 2, we list the band gaps calculated using the PBE and HSE06 functionals with and without the SOC effect for the cubic, tetragonal, and monoclinic phases of K_2_SnX_6_ (X = I, Br, Cl). As for other insulating compounds, the HSE06 calculations were found to predict wider band gaps compared with the PBE calculations. As shown in Fig. 1, when considering the SOC effect, the valence (conduction) bands were found to be pushed up (down) slightly in comparison with those without the SOC effect, resulting in narrower band gaps for all the phases of K_2_SnX_6_. It should be noted that the SOC effect becomes weaker on going from X = I to X = Cl, as the difference between the band gaps calculated with and without the SOC effect becomes smaller for all the phases. By considering the fact that HSE06 + SOC calculations can provide reasonable band gaps in good accordance with experiments, it can be said that K_2_SnI_6_ in the monoclinic phase and K_2_SnBr_6_ in the cubic phase are suitable for applications as light absorbers due to their appropriate band gaps of 1.16 and 1.65 eV calculated by the HSE06 + SOC method. On the other hand, the band gaps of K_2_SnCl_6_ in the cubic, tetragonal, and monoclinic phases were estimated by the HSE06 + SOC method to be 3.36, 3.49 and 4.04 eV respectively, implying that the chlorine-based double perovskites are not applicable for light absorbers but might be appropriate for charge carrier conducting materials. Meanwhile, for the cubic and tetragonal phases of the K_2_SnI_6_ compound, the HSE06 + SOC calculations yielded smaller band gaps of 0.31 and 0.74 eV, indicating that these phases could be useful for applications in infrared emitting diodes.

**Table 2:** Effective masses of electrons 
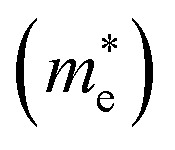
 and holes 
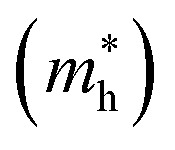
, reduced masses 
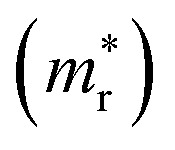
, static and high-frequency dielectric constants (*ε*_0_ and *ε*_∞_), exciton binding energies calculated using the static (*E*_b_) and high-frequency (*Ẽ*_b_) dielectric constants, and band gaps (*E*_g_) for K_2_SnX_6_ (X = Cl, Br, I), calculated with the PBE and HSE06 functionals with and without the SOC effect

Material	Phase	PBE (*m*_e_)			PBE		PBE (meV)		*E*_g_ (eV)			
		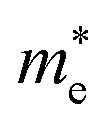	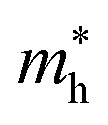	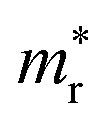	*ε*_∞_	*ε*_0_	*Ẽ*_b_	*E*_b_	PBE	PBE + SOC	HSE06	HSE06 + SOC
K_2_SnCl_6_	Cub.	0.47	0.99	0.32	2.69	6.28	599.9	110.1	2.41	2.40	3.43	3.36
	Tet.	0.50	1.06	0.34	2.71	6.45	632.0	111.6	2.45	2.44	3.51	3.49
	Mono.	1.01	1.69	0.63	2.75	8.33	1300.8	123.8	2.96	2.93	4.05	4.04
K_2_SnBr_6_	Cub.	0.33	0.83	0.24	3.29	7.36	297.5	59.4	1.01	0.92	1.81	1.65
	Tet.	0.46	0.83	0.29	3.48	11.45	330.6	30.6	1.56	1.40	2.50	2.32
	Mono.	0.72	1.15	0.44	3.86	12.52	403.5	38.3	1.83	1.77	2.68	2.57
K_2_SnI_6_	Cub.	0.17	0.46	0.12	4.95	13.76	68.4	8.9	0.05	0.04	0.52	0.31
	Tet.	0.39	0.69	0.25	5.54	15.41	108.4	14.0	0.44	0.32	0.96	0.74
	Mono.	0.58	0.78	0.33	9.59	17.20	104.0	15.3	0.81	0.69	1.40	1.16

**Fig. 2:**
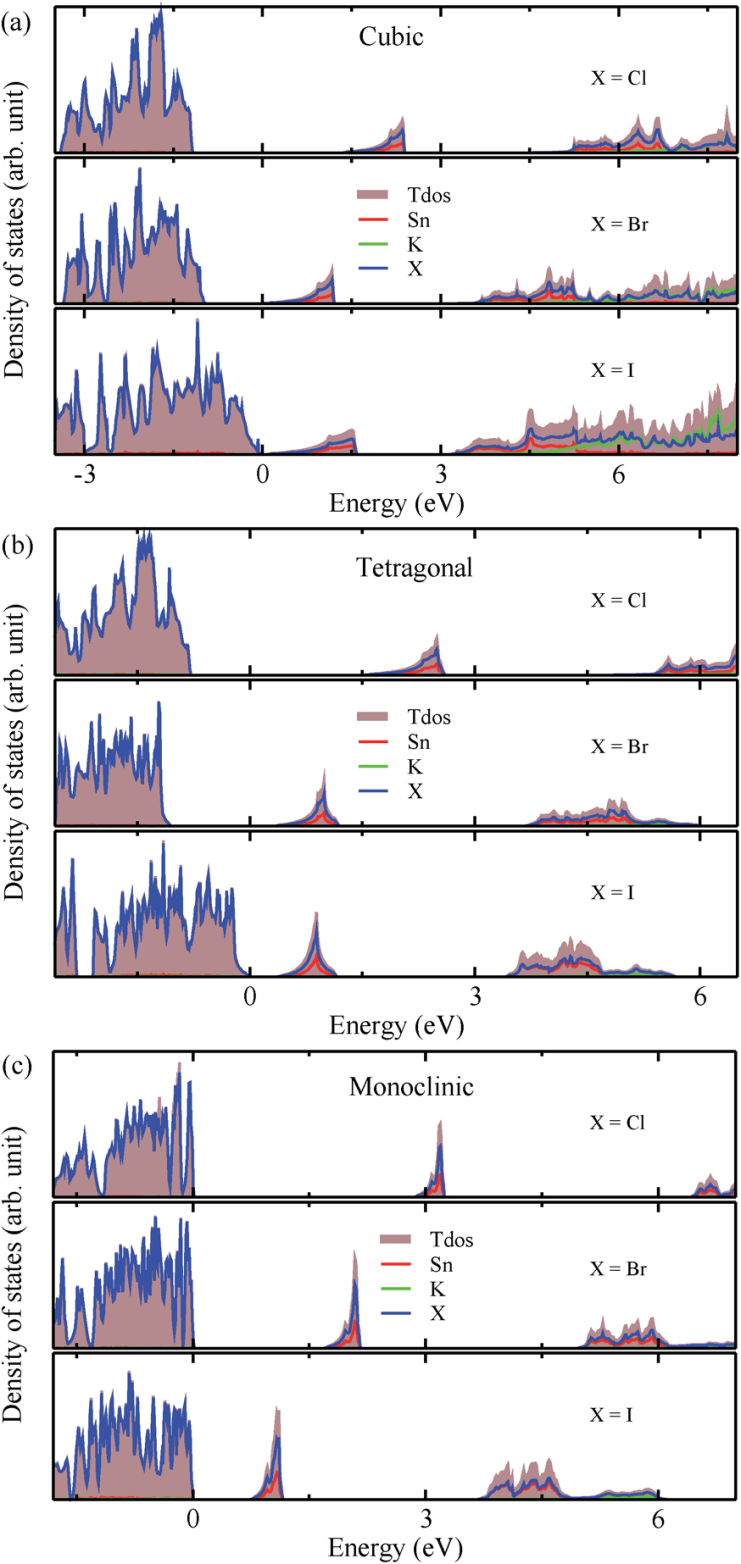
Total and atomic resolved electronic density of states in K_2_SnX_6_ (X = I, Br, Cl) in (a) cubic, (b) tetragonal and (c) monoclinic phases, calculated using the HSE functional with the SOC effect.

**Fig. 3:**
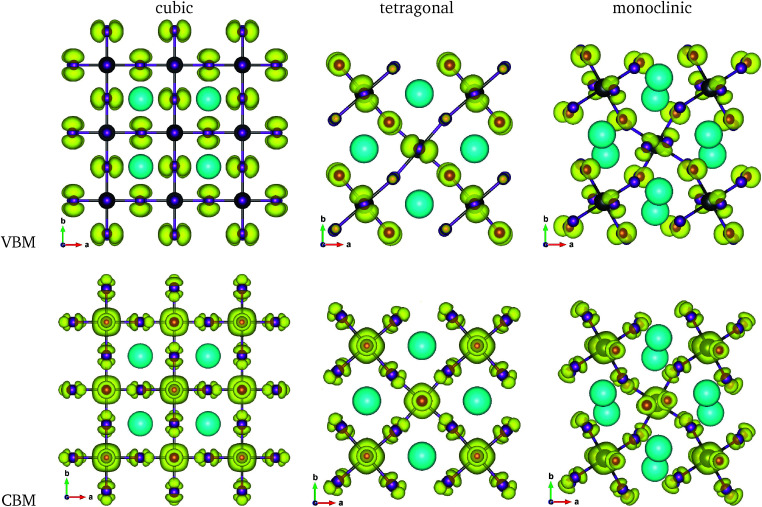
Isosurface plots of charge density corresponding to the valence band maximum (VBM) and conduction band minimum (CBM) at the value of 0.02 |e| Å^−3^ in K_2_SnI_6_ in the cubic, tetragonal, and monoclinic phases. Grey, purple and green balls represent Sn, I and K atoms.

The calculated band gaps display a distinct variation tendency with respect to the choice of halogen atom, such that for all the phases the band gaps systematically decrease as the ionic radius of the halogen anion increases. This variation tendency agrees well with previous calculations for hybrid organic–inorganic, all-inorganic, and vacancy-ordered double perovskites,^18,21,44,52,54^ which can be understood through the analysis of the total and atomic resolved DOS (see Fig. 2). As can be seen in Fig. 3, the VBM is derived from the p orbitals of the halide anion, while the CBM is characterized by antibonding between the Sn s and the halide p orbitals. Therefore, as the ionic radius of the halide anion increases and its electronegativity decreases on going from Cl to I, the VBM becomes higher while the CBM becomes lower, resulting in a decrease in the band gap.^18,54^ It should be noted that lowering the symmetry from cubic to monoclinic increases the band gap for all the K_2_SnX_6_ (X = I, Br, Cl) compounds, which correlates with the fact that as the symmetry lowers, the degree of octahedral distortion increases, resulting in a decrease in bonding strength between the neighboring halide anions, and thus a narrowing of the valence bands and an increase in the band gap.

### 3.3 Optical properties

In the next step, we further investigated various optical properties of the K_2_SnX_6_ (X = I, Br, Cl) compounds, including the effective masses of electrons and holes, static and high-frequency dielectric constants, exciton binding energies, and light absorption coefficients by use of the PBE functional, so as to assess their potential for use as light absorbers (Table 2). The effective masses of electrons and holes were observed to have a variation tendency similar to that of the band gap. That is, for a given compound, the effective masses of electrons and holes increase on moving from the cubic to the monoclinic phase, whereas for a given phase they decrease on going from Cl to I. This clearly indicates that on changing from a lower to a higher symmetry phase as well as going from X = Cl to X = I, K_2_SnX_6_ is expected to possess a higher mobility of charge carriers owing to the smaller effective masses of electrons and holes. In addition, as confirmed for other types of double perovskite,^44,54^ the effective masses of holes were found to be larger than those of electrons for all the phases of K_2_SnX_6_ (X = I, Br, Cl). Our calculated effective masses of 
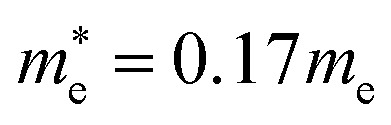
 and 
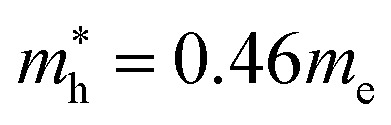
 for cubic K_2_SnI_6_ are smaller than the previously calculated values of 
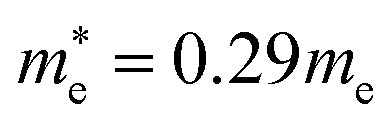
 and 
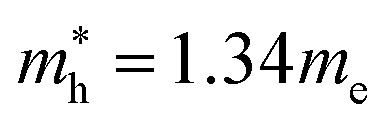
 for cubic Rb_2_SnI_6_, and 
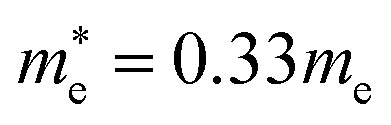
 and 
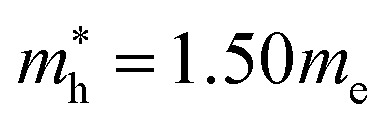
 for cubic Cs_2_SnI_6_.^54^ This agrees well with Cai’s report,^54^ which concluded that reducing the size of the A-site cation leads to a decrease in effective mass of both electrons and holes for A_2_BX_6_.

We next consider the dielectric constant which plays an important role in the assessment of optical properties. In this work, we calculated two kinds of dielectric constants, the high-frequency (*ε*_∞_) and static (*ε*_0_) dielectric constants, in which the former were extracted from frequency-dependent macroscopic dielectric functions calculated using the DFPT approach and the latter were estimated by post-processing the phonon dispersion properties. The calculated dielectric constants were shown to have a variation tendency slightly different to those of the band gaps and effective masses. For a given perovskite compound, the dielectric constants increase on going from the cubic to the monoclinic phase in accordance with the cases of band gap and effective masses, whereas for a given symmetry, they decrease on reducing the ionic radius of the halogen anion contrary to the former cases. It was eventually found that both lowering the symmetry and increasing the ionic radius of the halogen anion increase the dielectric constants for K_2_SnX_6_ (X = I, Br, Cl).

By use of the calculated effective masses of charge carriers and dielectric constants, we obtained exciton binding energies, which play a key role in discriminating whether electrons and holes behave as bound excitons or free charge carriers. In Table 2, we list two types of exciton binding energies, *Ẽ*_b_ and *E*_b_, which are calculated using the high-frequency (*ε*_∞_) and static (*ε*_0_) dielectric constants, respectively. It is clear that *E*_b_ is reduced by a factor of at least 6 compared to *Ẽ*_b_, because when the static dielectric constant is used, phonon processes contribute to screening the electrostatic interactions between electrons and holes, subsequently weakening their binding energies. For the cubic, tetragonal, and monoclinic phases of K_2_SnI_6_, the exciton binding energies *E*_b_ were estimated to be 8.9, 14.0, and 15.3 meV, respectively, which are obviously smaller than the values of 45–50 meV for cubic MAPbI_3_,^18,21^ while *E*_b_ of K_2_SnCl_6_ was determined to be about 2 times larger than the value for cubic MAPbI_3_. In the end, we emphasize that the calculated exciton binding energies have the same variation tendency as the band gap and effective masses, according to changes in phase symmetry and the size of the halogen anion. Light absorption coefficients were obtained using the calculated frequency-dependent dielectric constants, showing that the absorption onset gradually shifts to the blue region as the size of the halide anion decreases for a given phase, and as the symmetry of the phase lowers for a given compound (see Fig. S2†).

### 3.4 Phase stability

**Fig. 4:**
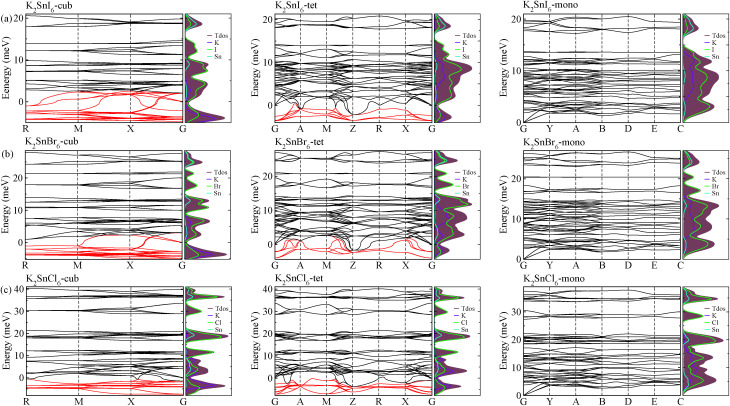
Phonon dispersion curves and atomic resolved density of states in (a) K_2_SnI_6_, (b) K_2_SnBr_6_ and (c) K_2_SnCl_6_ in the cubic (left), tetragonal (middle) and monoclinic (right) phases. Red lines show the anharmonic phonon modes with negative phonon energies.

As a final step, we investigated phonon dispersion with phonon total and atomic resolved DOS for the cubic, tetragonal, and monoclinic phases of K_2_SnX_6_ (X = I, Br, Cl) using the DFPT method with the PBE functional without the SOC effect. As shown in Fig. 4, the phonon dispersion curves of the cubic and tetragonal phases showed anharmonic phonon modes with negative phonon energies, indicating their dynamic instability at low temperature, whereas the monoclinic phases did not exhibit anharmonic features, indicating that K_2_SnX_6_ (X = I, Br, Cl) can stabilize in the monoclinic phase at low temperature. It is clear from the atomic resolved phonon DOS that as K and X atoms contribute to the phonon DOS corresponding to the anharmonic phonon modes, the dynamic instability in the cubic and tetragonal phases can be attributed to the vibrations of K and X atoms.

**Fig. 5:**
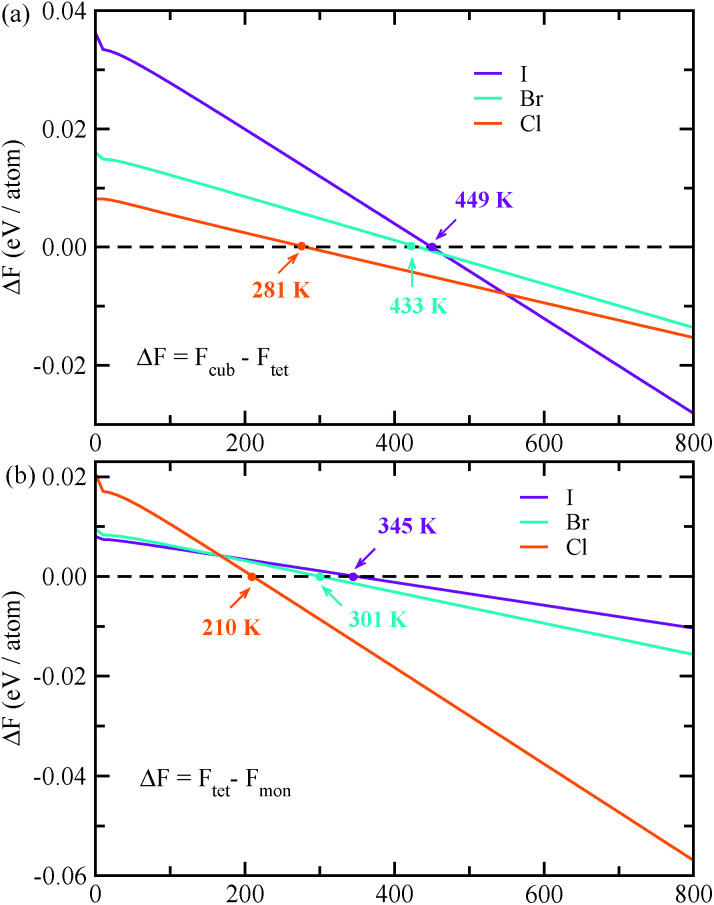
Helmholtz free energy differences between (a) cubic and tetragonal phases, and (b) tetragonal and monoclinic phases in K_2_SnX_6_ (X = I, Br, Cl).

By post-processing the phonon DOS, we finally calculated the constant-volume Helmholtz free energies of the cubic, tetragonal, and monoclinic phases for K_2_SnX_6_ (X = I, Br, Cl) on increasing the temperature from 0 to 1000 K with intervals of 10 K. The phase transition temperatures were estimated from the free energy differences between the cubic and tetragonal, and the tetragonal and monoclinic phases. As can be seen in Fig. 5, upon decreasing the temperature, K_2_SnX_6_ undergoes a phase transition from the cubic to the tetragonal phase at 449, 433 and 281 K, and from the tetragonal to the monoclinic phase at 345, 301 and 210 K for X = I, Br and Cl, respectively. From a previous experimental study on K_2_SnCl_6_,^46^ the phase transition temperatures for the cubic to tetragonal phase transition and the tetragonal to monoclinic phase transition were observed to be 262 and 255 K respectively, which are slightly different with our predicted values of 281 and 210 K. It should be noted that such deviations might stem from ignoring the volume change and the contributions of anharmonic modes to the phonon DOS in the calculation of Helmholtz free energies.

## 4 Conclusions

In conclusion, we have performed first-principles calculations to predict the structural, electronic, and optical properties, and the phase stabilities of the vacancy-ordered double perovskites K_2_SnX_6_ (X = I, Br, Cl) in the cubic, tetragonal, and monoclinic phases. Our calculations reveal that the energy band gaps, effective masses of electrons and holes, and exciton binding energies increase as the symmetry of the phase lowers for a given compound and as the size of the halogen anion increases for a given phase. In particular, the band gaps and exciton binding energies of cubic K_2_SnBr_6_ and monoclinic K_2_SnI_6_ were calculated to be 1.65 eV and 59.4 meV for the former case and 1.16 eV and 15.3 meV for the latter case, providing a conclusion that on account of their suitable band gaps and optical properties, cubic K_2_SnBr_6_ and monoclinic K_2_SnI_6_ are promising candidates for light absorber materials in PSCs. Through the phonon calculations, the cubic and tetragonal phases were found to exhibit anharmonic phonon modes, whereas the monoclinic phases did not, and these anharmonic features were attributed to vibrations of K and X atoms as identified by the atomic resolved phonon DOS. Finally, we calculated the Helmholtz free energy differences between the cubic and tetragonal phases, and the tetragonal and monoclinic phases, giving phase transition temperatures of 449, 433 and 281 K for the cubic–tetragonal transition, and 345, 301 and 210 K for the tetragonal–monoclinic transition for X = I, Br and Cl, respectively. Our calculations provide a comprehensive understanding of the material properties of the vacancy-ordered double perovskites K_2_SnX_6_, and could be helpful in devising low-cost and high performance PSCs.

## Conflicts of interest

There are no conflicts to declare.

## Supplementary Material

RA-010-C9RA09232C-s001
