# Supplementary material for: Computational prediction of structural, electronic, and optical properties and phase stability of double perovskites K2SnX6 (X = I, Br, Cl)
Source: RSC Adv. 2019 Dec 24;10(1):201–9. doi: 10.1039/c9ra09232c (PMC9048279; doi:10.1039/c9ra09232c)
Supplement: RA-010-C9RA09232C-s001 [file RA-010-C9RA09232C-s001.pdf]

Electronic Supplementary Material (ESI) for RSC Advances.
This journal is © The Royal Society of Chemistry 2019

Supporting Information – Computational Prediction of Structural, Electronic, Optical Properties and Phase Stability of Double Perovskites K_2SnX_6 (X = I, Br, Cl)

Un-Gi Jong[a,b]*, Chol-Jun Yu[a]†, Yun-Hyok Kye[a,b]

[a] *Chair of Computational Materials Design, Faculty of Materials Science, Kim Il Sung University, Ryongnam-Dong, Taesong District, Pyongyang, Democratic People's Republic of Korea*

[b] *Natural Science Centre, Kim Il Sung University, Ryongnam-Dong, Taesong District, Pyongyang, Democratic People's Republic of Korea*

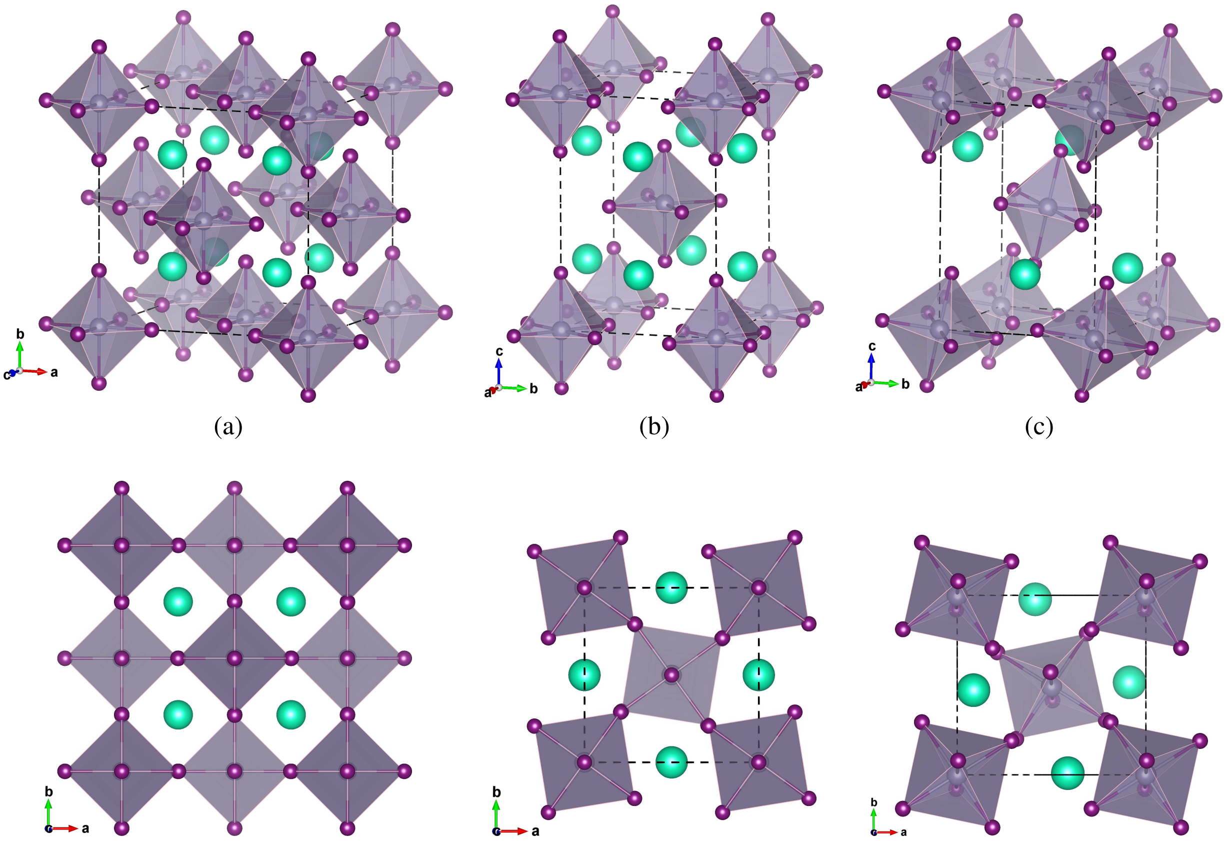

Figure S1. Polyhedral view of optimized crystal structures of (a), (d) cubic, (b), (e) tetragonal, and (c), (f) monoclinic K_2SnX_6 in perspective (top panel) and $x-y$ plane (lower panel) . Grey, purple and green balls represent Sn, X and K atoms, respectively.

*Corresponding author: Un-Gi Jong, Email: ug.jong@ryongnamsan.edu.kp
†Corresponding author: Chol-Jun Yu, Email: cj.yu@ryongnamsan.edu.kp

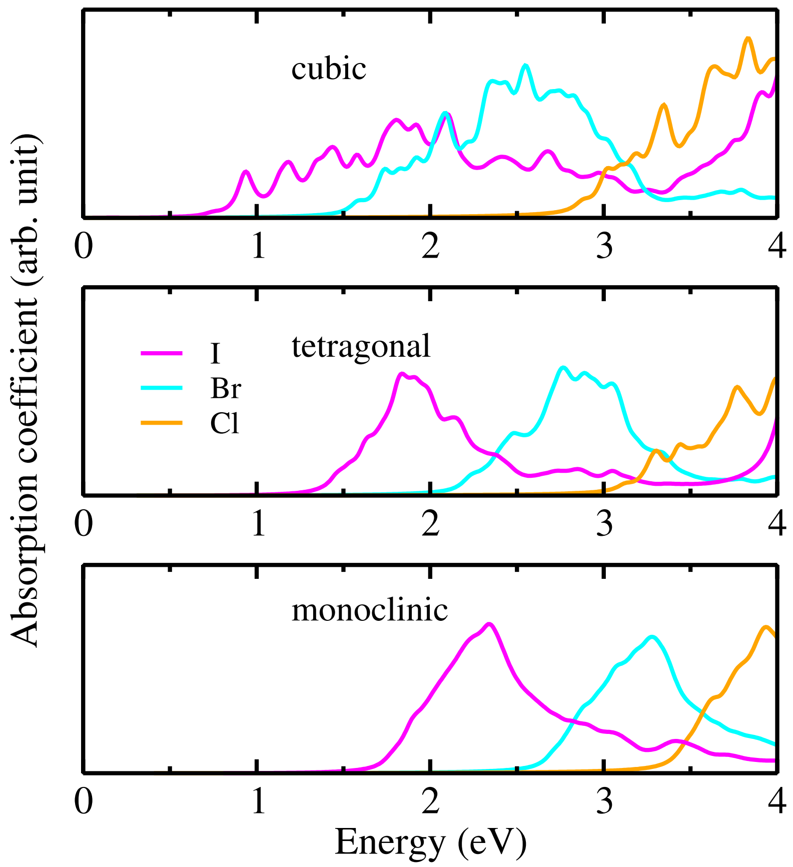

Figure S2. Light-absorption coefficients calculated within the density functional perturbation theory for the cubic, tetragonal, and monoclinic phases of K_2SnX_6 (X = I, Br, Cl).

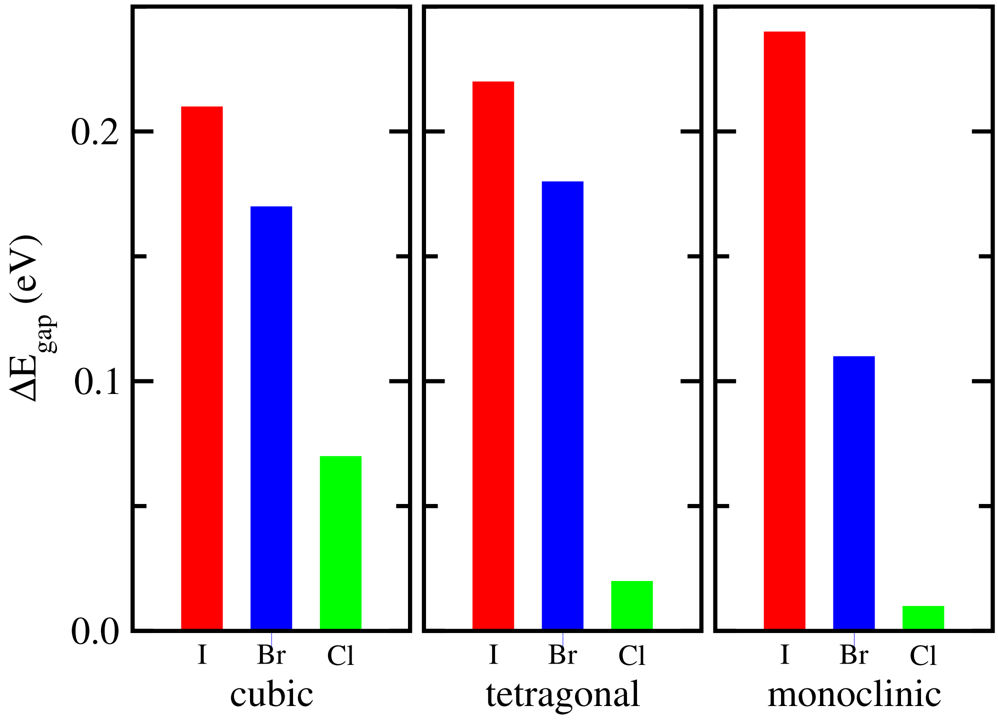

Figure S3. Band gap differences between HSE and HSE+SOC calculations for the cubic, tetragonal, and monoclinic phases of K_2SnX_6 (X = I, Br, Cl).
